# Therapeutically useful mycobacteriophages BPs and Muddy require trehalose polyphleates

**DOI:** 10.1038/s41564-023-01451-6

**Published:** 2023-08-24

**Authors:** Katherine S. Wetzel, Morgane Illouz, Lawrence Abad, Haley G. Aull, Daniel A. Russell, Rebecca A. Garlena, Madison Cristinziano, Silke Malmsheimer, Christian Chalut, Graham F. Hatfull, Laurent Kremer

**Affiliations:** 1 Department of Biological Sciences, University of Pittsburgh, Pittsburgh, PA USA; 2 Centre National de la Recherche Scientifique UMR 9004, Institut de Recherche en Infectiologie de Montpellier (IRIM), Université de Montpellier, Montpellier, France; 3 Institut de Pharmacologie et de Biologie Structurale, Université de Toulouse, CNRS, UPS, Toulouse, France; 4 INSERM, IRIM, Montpellier, France

**Keywords:** Bacteriophages, Bacterial genetics, Phage biology

## Abstract

Mycobacteriophages show promise as therapeutic agents for non-tuberculous mycobacterium infections. However, little is known about phage recognition of *Mycobacterium* cell surfaces or mechanisms of phage resistance. We show here that trehalose polyphleates (TPPs)—high-molecular-weight, surface-exposed glycolipids found in some mycobacterial species—are required for infection of *Mycobacterium abscessus* and *Mycobacterium smegmatis* by clinically useful phages BPs and Muddy. TPP loss leads to defects in adsorption and infection and confers resistance. Transposon mutagenesis shows that TPP disruption is the primary mechanism for phage resistance. Spontaneous phage resistance occurs through TPP loss by mutation, and some *M. abscessus* clinical isolates are naturally phage-insensitive due to TPP synthesis gene mutations. Both BPs and Muddy become TPP-independent through single amino acid substitutions in their tail spike proteins, and *M. abscessus* mutants resistant to TPP-independent phages reveal additional resistance mechanisms. Clinical use of BPs and Muddy TPP-independent mutants should preempt phage resistance caused by TPP loss.

## Abstract (web summary)

Trehalose polyphleates are surface-exposed glycolipids that are required for successful infection by phages BPs and Muddy when infecting *Mycobacterium abscessus* and *M. smegmatis*.

## Main

Non-tuberculous mycobacteria include several important human pathogens such as *Mycobacterium abscessus* and *M. avium*^1,2^. These infections are often refractory to effective antibiotic treatment due to both intrinsic and acquired resistance mutations, and new treatment options are needed^3^. The therapeutic application of mycobacteriophages shows some promise for the treatment of pulmonary infections in persons with cystic fibrosis^4–6^, disseminated infection following bilateral lung transplantation^4^ and disseminated *M. chelonae* infection^7^. However, broadening therapy beyond single-patient compassionate use applications will require expansion of the repertoire of therapeutically useful phages and increasing host range such that a higher proportion of clinical isolates can be treated^5,8,9^. Clinical administration of bacteriophages is anticipated to give rise to phage-resistant mutants and disease recurrence^10^, but the frequency and mechanisms of mycobacteriophage resistance are poorly understood^11^. Very few mycobacteriophage receptors are known, although glycopeptidolipids (GPLs) are proposed as receptors for mycobacteriophage I3 in *M. smegmatis*^12^.

Over 12,000 individual mycobacteriophages have been described, with most having been isolated on *M. smegmatis*^13^. The genome sequences of 2,200 of these show them to be highly diverse genetically and pervasively mosaic^14,15^. They can be sorted into groups of genomically related phages (for example, Cluster A, B, C and so on), some of which can be readily divided into subclusters (for example, Subcluster A1, A2, A3 and so on) on the basis of sequence variation^16,17^. Seven of the sequenced phages currently have no close relatives and are designated as ‘singletons’^18^. A subset of these phages have relatively broad host range and are also able to efficiently infect *M. tuberculosis*, including phages in Clusters/Subclusters A2, A3, G1, K1, K2, K3, K4 and AB^19,20^. A similar subset of phages also infect some clinical isolates of *M. abscessus*, although it is noteworthy that phage host ranges on these strains are highly variable (even for related phages within clusters/subclusters) and are highly variable among different clinical isolates^4,9^. There is also substantial variation in the outcomes of phage infection of *M. abscessus* strains, with notable differences between rough and smooth colony morphotypes^9^. For example, a smaller proportion of smooth isolates are susceptible to phage infection compared with rough strains, as determined by plaque formation, and none of the smooth strains is efficiently killed by any phage tested^9^.

Mycobacterial cell walls characteristically have a mycolic acid-rich outer layer referred to as the mycobacterial outer membrane or mycomembrane^21^. In addition to abundant mycolic acids, there are numerous other types of complex molecule including multiple acylated lipids such as di- and polyacyltrehalose (DAT and PAT), phthiocerol dimycocerosate and sulfoglycolipids, although not all are found in all *Mycobacterium* species. Smooth strains of *M. abscessus* have abundant GPLs, whereas these are lacking or greatly less abundant in rough strains^22,23^. Recently, it has been shown that some mycobacterial species, including *M. abscessus*, have trehalose polyphleates (TPPs), which are high-molecular-weight, surface-exposed glycolipids, in their cell walls^24,25^. These TPPs may be important for *M. abscessus* virulence and are associated with clumping and cording^25^. A five-gene cluster, including a polyketide synthetase (Pks), is required for TPP biosynthesis and TPP precursor (DAT) transport to the outer surface of the cell by MmpL10 (ref. ^26^). TPPs are not present in *M. tuberculosis* although DAT and PAT are^26^. The specific roles of TPPs are not known, but their position on the outer surface makes them candidates for use as phage receptors.

Here we show that TPPs are required for the binding and infection of *M. abscessus* by phages BPs and Muddy. These phages share little or no nucleotide similarity but both have been used therapeutically, sometimes in combination with each other^4,27^. *M. abscessus* transposon insertion mutants that are resistant to these phages map in all five genes involved in TPP synthesis, all have lost TPPs from their cell walls and phage adsorption is lost. Spontaneous phage-resistant mutants of some *M. abscessus* clinical isolates also have mutations in the known TPP synthesis genes, and some *M. abscessus* clinical isolates that are insensitive to BPs and Muddy are naturally defective in TPPs. However, the TPP requirement can be readily overcome by mutations in phage tail spike proteins, suggesting that TPPs are acting as a co-receptor, and the cell wall binding target of the phages is probably essential for mycobacterial viability. *M. abscessus* strains resistant to BPs and Muddy TPP-independent mutants reveal new mechanisms of phage resistance.

## Results

### Transposon mutagenesis of *M. abscessus* clinical isolates

**Fig. 1:**
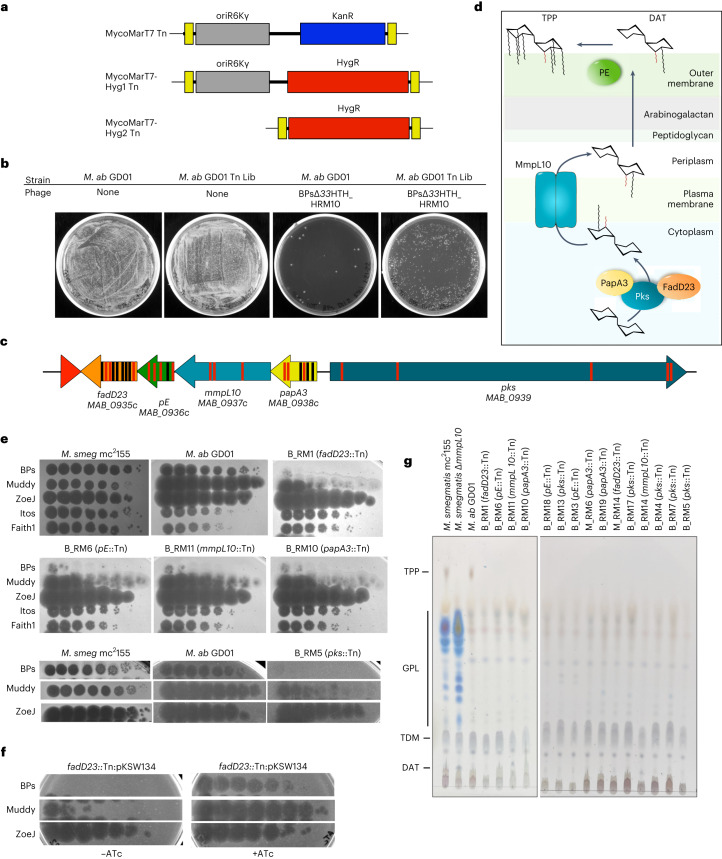
Identification of phage-resistant transposon insertion mutants of *M. abscessus* GD01. **a**, Construction of MycoMarT7-Hyg1 and MycoMarT7-Hyg2. Transposon delivery phage phiMycoMarT7 delivers a transposon containing *Escherichia coli* ori6Kγ (grey) and a kanamycin resistance cassette (blue), flanked by inverted repeats (yellow boxes). CRISPY-BRED^31^ was used to create phiMycoMarT7-Hyg1 and phiMycoMarT7-Hyg2, which deliver transposons containing oriR6K (grey box) and a hygromycin resistance cassette (red box), or only a hygromycin resistance cassette. **b**, *M. abscessus* GD01 or a transposon library of *M. abscessus* strain GD01 (*M.* *ab* GD01 Tn Lib) was plated on solid media or solid media seeded with phage BPs_∆*33*HTH_HRM10. **c**, Locations of transposon insertions in the TPP locus in phage-resistant mutants. Red and black bars show the locations of insertions in strains isolated as resistant to BPs∆*33*HTH_HRM10 and Muddy, respectively. **d**, Proposed roles of Pks, PapA3, FadD23, MmpL10 and PE in the synthesis and transport of TPPs and DAT. **e**, Tenfold serial dilutions of phages were spotted onto solid media with *M. smegmatis* mc^2^155, *M. abscessus* GD01 or representative *M. abscessus* GD01 transposon insertion mutant strains: GD01Tn_BPs_HRM10_RM1 (B_RM1); GD01Tn_BPs_HRM10_RM6 (B_RM6); GD01Tn_BPs_HRM10_RM11 (B_RM11); GD01Tn_BPs_HRM10_RM10 (B_RM 10); GD01Tn_BPs_HRM10_RM5 (B_RM 5). The locations of Tn insertions are indicated in parentheses. Phages used are: BPs∆*33*HTH_HRM10 (‘BPs’), Muddy, ZoeJΔ*43–45* (‘ZoeJ’), Itos and Faith1Δ*38–40* (‘Faith1’). Plaque assays were performed at least twice with similar results. **f**, Tenfold serial dilutions of phages were spotted onto solid media with strain GD01 *fadD23*::Tn (GD01Tn_BPs_HRM10_RM1) containing plasmid pKSW134 with gene *fadD23* under expression of an ATc-inducible promoter. FadD23 is not expressed in the absence of ATc (left panel) but is induced by ATc (right panel). Plaque assays were performed at least twice with similar results. **g**, Thin-layer chromatography (TLC) analysis of total lipids extracted from *M. abscessus* GD01 and mutants with transposon insertions in the TPP synthesis and transport genes. *M. smegmatis* mc^2^155 and a Δ*mmpL10* mutant strain of *M. smegmatis* are also included as controls.

*M. abscessus* GD01 (subspecies *massiliense*) was selected for transposon mutagenesis as it is the first clinical isolate treated therapeutically^4^ and is killed well by phages Muddy, ZoeJΔ*45* and BPsΔ*33*HTH_HRM10, mapping in Clusters AB, K2 and G1, respectively. Muddy is a lytic phage and ZoeJΔ*45* and BPsΔ*33*HTH_HRM10 are engineered lytic derivatives of ZoeJ^28^ and BPs^29^, respectively. Because GD01, similar to many *M. abscessus* isolates, is kanamycin resistant (minimum inhibitory concentration (MIC) > 128 µg ml^−1^)^30^, we re-engineered the extant Kan^R^ MycoMarT7 transposon using CRISPY-BRED^31^ to include an Hyg^R^ cassette, constructing derivatives both with and without the existing R6Kγ origin of replication (Fig. 1a). The shorter transposon (MycoMarT7-Hyg2) transduced strain GD01 ~100 times more efficiently than the longer MycoMarT7-Hyg1 transposon; this efficiency difference was not observed for *M. smegmatis*. We transduced strain GD01 with MycoMarT7-Hyg2 and selected Hyg-resistant transductants on solid media to yield a random mutagenesis library (Fig. 1b). We note that the parent of the transposon delivery phages, TM4, does not form plaques on any *M. abscessus* strain^9^ but efficiently delivers DNA to *M. abscessus* cells^32^.

### Phage-resistant mutants are defective in TPPs

**Extended Data Fig. 1:**
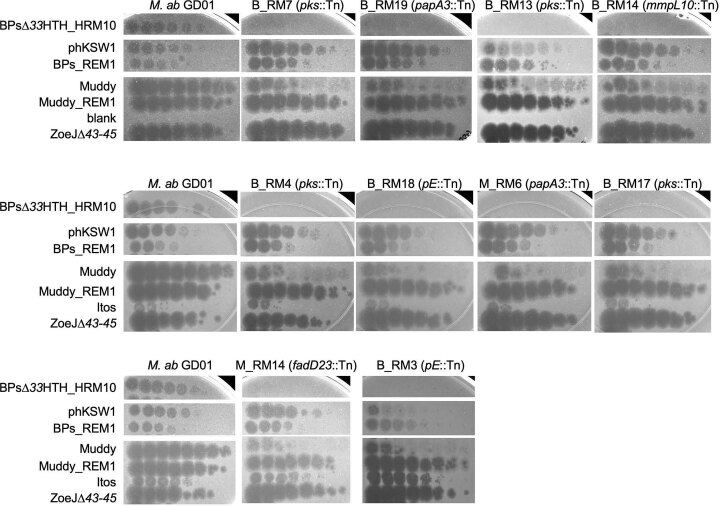
Plaque assays of phages on *M. abscessus* GD01 transposon insertion mutants. Phages as shown on the left were spotted onto solid media with *M. abscessus* GD01 or transposon insertion mutant strains. Each row indicates a set strains tested together.

To identify *M. abscessus* GD01 phage-resistant mutants, the Tn library was plated on solid media seeded with either BPsΔ*33*HTH_HRM10 or Muddy. Single colonies were recovered at a frequency of ~10^−^^3^ and 20 individual colonies were picked from each selection, rescreened and characterized (Fig. 1, Extended Data Fig. 1 and Extended Data Table 1). Eighteen of the 20 BPsΔ*33*HTH_HRM10-resistant candidates were mapped, all of which have transposon insertions in a gene cluster involved in TPP synthesis; some appear to have secondary transposon insertions mapping elsewhere (Extended Data Table 1 and Fig. 1c,d). Thirteen of the 20 Muddy resistant candidates were mapped and surprisingly, all also contain insertions in TPP synthesis genes (Extended Data Table 1 and Fig. 1c,d). TPP synthesis has previously been reported to be non-essential^33^ and these observations suggest that loss of TPPs is the primary mechanism of resistance to both BPsΔ*33*HTH_HRM10 and Muddy. Further analysis showed that all of the mutants tested have similar phenotypes, with a large reduction in the efficiency of plaquing of BPsΔ*33*HTH_HRM10 and a more modest reduction in the efficiency of plaquing of Muddy, but with formation of very turbid plaques (Fig. 1e). Complementation of a *fadD23* Tn mutant confirmed that phage resistance results from TPP loss (Fig. 1f). All of the strains that we tested remain sensitive to ZoeJ∆*43–45*, Itos and Faith1∆*38–40* (Fig. 1e and Extended Data Fig. 1).

Analysis of cell wall lipids shows that all of the mutants tested have lost TPPs (Fig. 1g). Interruption of TPP precursor transport (as in an *mmpL10* mutant; Fig. 1d), or loss of PE protein needed for the final step of TPP synthesis (Fig. 1d) can result in accumulation of the DAT precursor^24,26^, and our *mmpL10* transposon insertion mutants did accumulate DAT. Our *pE* mutants did not accumulate DAT and the Tn insertions may be polar, interrupting *fadD23* expression and DAT synthesis (Fig. 1c,d,g). No defects in trehalose dimycolate synthesis were observed, trehalose dimycolate being transported by MmpL3 (ref. ^34^) (Fig. 1g).

### Phage BPs tail spike mutants are TPP independent

**Fig. 2:**
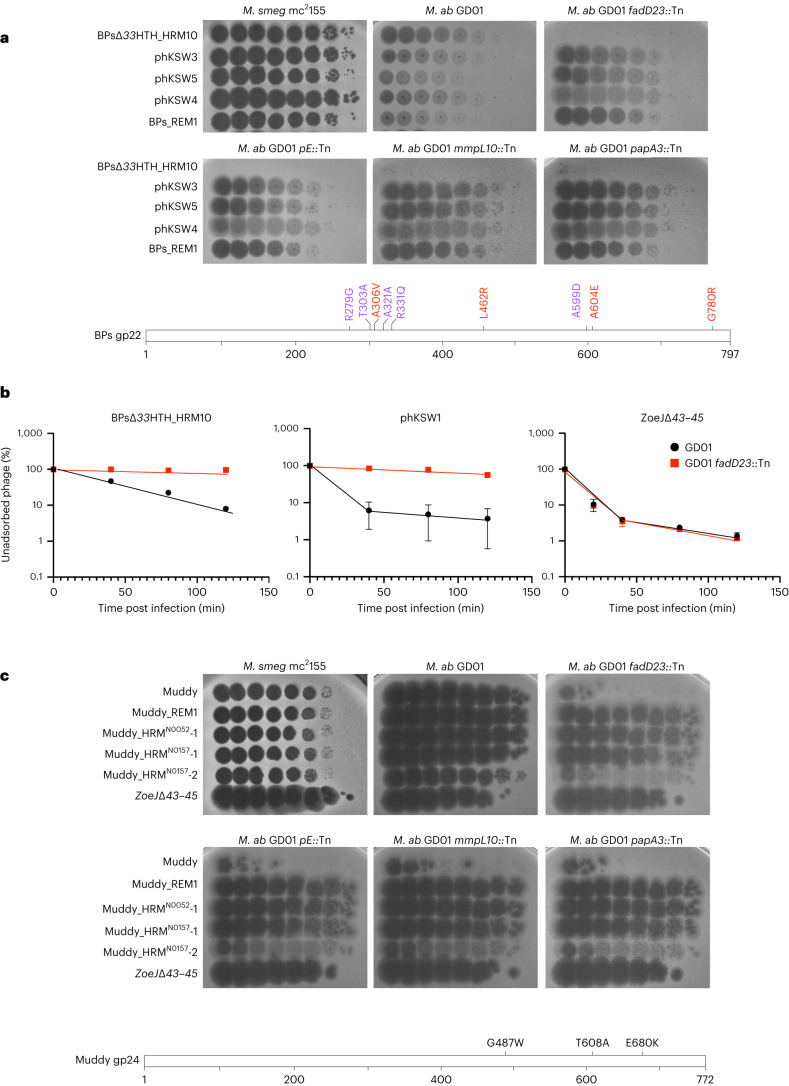
Mutants of BPs∆*33*HTH_HRM10 and Muddy overcome TPP loss. **a**, Tenfold serial dilutions of BPs∆*33*HTH_HRM10 and gp22 mutants (as indicated on the left; see Extended Data Table 2) were spotted onto solid media with *M. smegmatis* mc^2^155, *M. abscessus* GD01 or *M. abscessus* GD01 transposon insertion mutant strains. Plaque assays were performed at least twice with similar results. The locations of amino acid substitutions in BPs∆*33*HTH_HRM10 gp22 conferring the ability to infect TPP-deficient strains (red) or previously found to broaden host range to include *M. tuberculosis* (purple) (bottom panel) are indicated. The A306V and A604E substitutions were identified with both assays. **b**, Adsorption of phages BPs∆*33*HTH_HRM10, phKSW1 and ZoeJ∆*43–45* to *M. abscessus* strains GD01 and GD01 *fadD23*::Tn (GD01Tn_BPs_HRM10_RM1) as indicated by the percentage of unadsorbed phages remaining in infection supernatants at different times after infection. Assays were performed in duplicate twice and data presented are mean ± s.d. **c**, Tenfold serial dilutions of Muddy and Muddy gp24 mutants (as indicated on the left) were spotted onto solid media with *M. smegmatis* mc^2^155, *M. abscessus* GD01 or *M. abscessus* GD01 transposon insertion mutant strains. Plaque assays were performed at least twice with similar results. The locations of amino acid substitutions in Muddy gp24 that confer the ability to infect TPP-deficient strains (bottom panel) are indicated.

Although BPs∆*33*HTH_HRM10 does not efficiently infect *M. abscessus* TPP synthesis mutants, plaques were observed at high phage titres that are candidates for TPP-independent mutants (Fig. 1e). Five individual plaques were purified, shown to have heritable infection of *M. abscessus* TPP mutants and were further characterized. Two were isolated on *M. abscessus* GD01 *fadD23::Tn* (phKSW2 and phKSW3), two on GD01 *pE::Tn* (phKSW4 and phKSW5) and one on GD180_RM2 (BPs_REM1; see below); an additional mutant (phKSW1) was isolated on *M. smegmatis ∆MSMEG_5439* (Extended Data Table 2; see below). These mutants form clear plaques on all TPP synthesis pathway mutants tested (Fig. 2a), and sequencing showed that all have single amino acid substitutions in the predicted BPs tail spike protein, gp22 (Extended Data Table 2). Interestingly, two of these substitutions, gp22 A306V and A604E (present in phKSW3 and phKSW5, respectively), were reported previously as BPs host range mutants able to infect *M. tuberculosis*^19,29^. The gp22 A604E substitution is also present in phage BPsΔ*33*HTH_HRM^GD03^ that infects some other *M. abscessus* strains^4^. Although phKSW4 (and phKSW2; Extended Data Table 2) has a gp22 L462R substitution, BPs_REM1 has both a gp22 L462R substitution and a G780R substitution. BPs_REM1 forms somewhat clearer plaques than phKSW4 on the TPP mutants (Fig. 2a), suggesting that G780R has an additive effect towards clear plaque formation.

### TPPs are required for BPs adsorption to *M. abscessus* GD01

Because TPPs are surface exposed and are required for BPsΔ*33*HTH_HRM10 infection, we tested whether they are required for adsorption (Fig. 2b). Wild-type BPs adsorb relatively poorly to *M. smegmatis*^19^ and BPsΔ*33*HTH_HRM10 adsorption is similarly poor on *M. abscessus* GD01 (Fig. 2b). However, BPsΔ*33*HTH_HRM10 is clearly defective in adsorption to a GD01 *fadD23*::Tn mutant (Fig. 2b, left panel). Interestingly, the TPP-independent phage phKSW1 (Extended Data Table 2) adsorbs considerably faster to GD01 (as does a BPs gp22 A604E mutant in *M. smegmatis*^19^) than its parent phage (Fig. 2b, middle panel) and shows only a small improvement in adsorption to the GD01 *fadD23*::Tn mutant relative to BPsΔ*33*HTH_HRM10 infection of GD01 (Fig. 2b). In contrast, ZoeJΔ*43*–*45* adsorbs similarly to both *M. abscessus* strains (Fig. 2b, right panel).

### Phage Muddy tail spike mutants are also TPP independent

We similarly isolated a resistance escape mutant of Muddy (Muddy_REM1, Extended Data Table 2) and, together with three Muddy mutants with expanded *M. tuberculosis*^20^ host ranges, characterized their infection of TPP pathway mutants (Fig. 2c). Three of the mutants (Muddy_REM1, Muddy_HRM^N0157^-1 and Muddy_HRM^N0052^-1) efficiently infect all of the TPP pathway mutants; Muddy_HRM^N0157^-2 forms very turbid plaques on all of the mutants, similar to wild-type Muddy (Fig. 2c). Sequencing showed that Muddy_REM1 contains a single base substitution in the tail spike gene *24* conferring an E680K substitution (Extended Data Table 2), the same substitution as in Muddy_HRM^N0052^-1; Muddy_HRM^N0157^-1 and Muddy_HRM^N0157^-2 have G487W and T608A substitutions in gp24, respectively^20^.

### Loss of TPPs in spontaneous *M. abscessus* resistant mutants

**Fig. 3:**
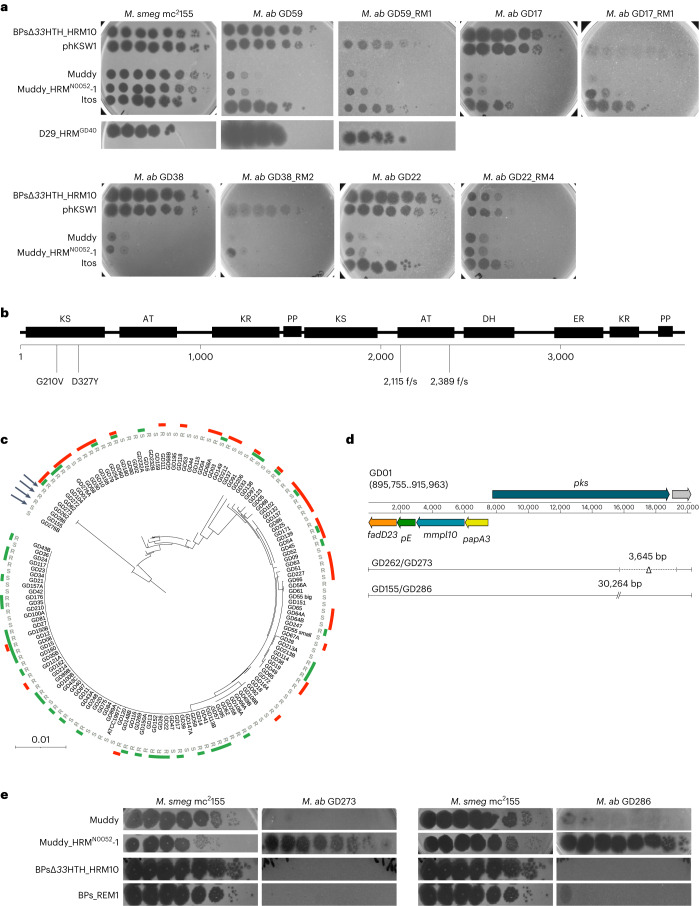
Phage infection profiles of *M. abscessus* phage-resistant mutants. **a**, Tenfold serial dilutions of phage lysates (as indicated on the left) were spotted onto solid media with *M. smegmatis* mc^2^155, the parent *M. abscessus* strains or spontaneously isolated phage-resistant mutant (RM) derivatives. Plaque assays were performed at least twice with similar results. **b**, A schematic representation of *M. abscessus* Pks showing the location of predicted functional domains and the amino acid changes in spontaneous phage-resistant mutants below. Domain abbreviations are: AT, acyltransferase; KS, ketosynthase, KR, ketoreductase; DH, dehydratase; ER, enoylreductase; PP, phosphopantetheinylate acyl carrier protein. Domains were identified using the PKS analysis web site at http://nrps.igs.umaryland.edu/ (ref. ^53^). **c**, Amino acid sequences from the five TPP synthesis pathway genes in 143 *M. abscessus* clinical isolates (and *M. abscessus* ATCC19977) were concatenated and used to construct a phylogenetic tree. Strain morphotypes are labelled as either rough (R) or smooth (S). Susceptibilities to phages BPs∆*33*HTH_HRM10 and Muddy are represented in green and red, respectively^9^. Arrows indicate strains in **d** and **e**. **d**, Position of large deletions (GD262 and GD273) or insertions (GD155 and GD286) in the *pks* gene with respect to the GD01 TPP locus. **e**, Tenfold serial dilutions of phage lysates (as indicated on the left) were spotted onto solid media with either *M. smegmatis* mc^2^155 or *M. abscessus* strains GD273 and GD286. Plaque assays were performed at least twice with similar results.

We previously reported *M. abscessus* mutants spontaneously resistant to BPs derivatives^9^. Two of the strains (GD17_RM1 and GD22_RM4, Extended Data Table 3) have mutations in *pks* and are at least partially resistant to BPsΔ*33*HTH_HRM10 (ref. ^9^). We have similarly isolated three additional spontaneous mutants resistant to BPsΔ*33*HTH_HRM10, two of which (GD38_RM2 and GD59_RM1) have mutations in *pks*; the third (GD180_RM2) has a nonsense mutation in *mmpL10* (Extended Data Table 3). BPsΔ*33*HTH_HRM10 does not form plaques on mutants GD38_RM2, GD17_RM1 or GD59_RM1 and forms very small plaques at a reduced efficiency of plaquing on GD22_RM4 (Fig. 3a). Thus, point mutations in *M. abscessus* TPP synthesis genes can give rise to BPs resistance, although these have not been observed clinically^5^. These mutants are infected well by other phages we tested that infect the parent strain (Fig. 3a).

The *M. abscessus* Pks protein (MAB_0939) is a 3,697-residue multidomain protein (Fig. 3b). Two of the spontaneously resistant mutants have frameshift mutations close to the midpoint of the gene (at codons 2,115 and 2,389, Extended Data Table 3 and Fig. 3b) and two others have amino acid substitutions in the N-terminal ketosynthase (KS) domain (Extended Data Table 3 and Fig. 3b). We note that the two frameshift mutations are in the second acyltransferase (AT) domain and leave the upstream domains intact (Fig. 3b).

### Some phage-insensitive clinical isolates lack TPPs

*M. abscessus* clinical isolates vary greatly in their sensitivity to BPs∆*33*HTH_HRM10 and Muddy^9^. There are probably numerous determining factors, but these could include loss of TPPs. Analysis of the TPP synthesis proteins (Pks, PE, PapA3, MmpL10 and FadD23) of 143 sequenced clinical isolates and reference strain ATCC19977 identified 37 distinct genotypes that generally correlate with global nucleotide similarity (Fig. 3c); however, no evident correlation between these variations and sensitivity to BPs∆*33*HTH_HRM10 and/or Muddy was observed (Fig. 3c). Most of the variations observed reflect amino acid substitutions, although two strains (GD262 and GD273) have identical large deletions in *pks* (3,645 bp) and two others (GD155 and GD286) have translocations resulting in 30.2 kbp insertions in *pks* (Fig. 3d). Both GD273 and GD286 have phage infection profiles consistent with TPP loss, and the TPP-independent mutant Muddy_HRM^N0052^-1 overcomes the defect (Fig. 3e). GD262, GD273 and GD286 are not susceptible to BPsΔ*33*HTH_HRM10 or the TPP-independent mutant BPs_REM1 (Fig. 3e), and these strains probably carry additional phage defence mechanisms targeting BPs and its derivatives. GD155 has a smooth colony morphotype (Fig. 3c) and is not susceptible to any of the phages tested here.

### Complementation restores TPP synthesis and phage infection

**Fig. 4:**
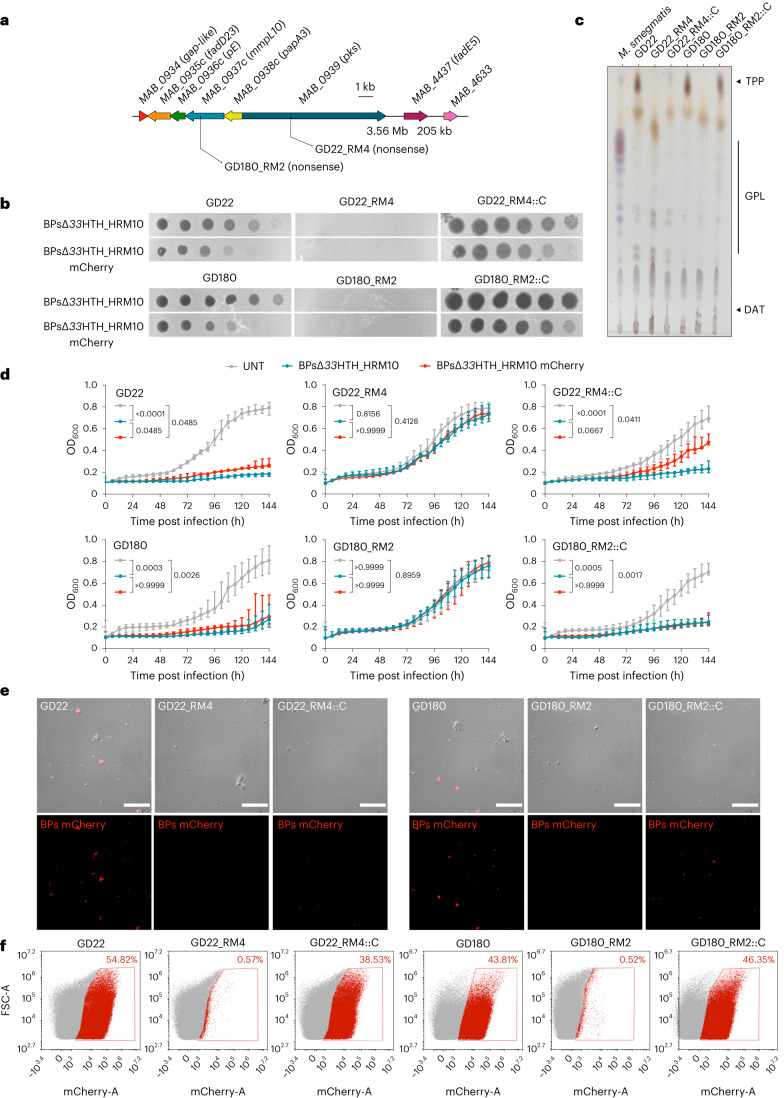
TPPs are essential for BPs∆*33*HTH_HRM10 to lyse *M. abscessus*. **a**, Representation of the *M. abscessus* TPP locus showing mutations affecting the clinical strains studied. **b**, Phages were spotted as tenfold serial dilutions onto clinical strains (GD22 and GD180), spontaneous resistant mutants (RM) and complemented strains (::C). Plates were incubated for 2–3 d at 37 °C before imaging. The assay was repeated at least three times and a representative experiment is shown. **c**, TLC analysis of total lipids extracted from *M. abscessus* clinical strains, resistant mutants and complemented strains. Eluent: CHCl_3_/CH_3_OH (90:10 v/v). Anthrone was sprayed on the plates to visualize the lipid profile, followed by charring. **d**, Liquid growth of the strains with BPs∆*33*HTH_HRM10 or BPs∆*33*HTH_HRM10 mCherry (MOI 10) or without phage (untreated; UNT) monitored every 6 h for 6 d at 37 °C in 7H9/OADC supplemented with 1 mM CaCl_2_. Data are plotted as the median ± interquartile range of three independent experiments done in triplicate. Statistical analysis conducted to compare the differences at 144 h between strains was done with a two-sided Dunn’s multiple comparisons test, with *P* values indicated. **e**, Representative fields of *M. abscessus* clinical strains infected with BPs∆*33*HTH_HRM10 mCherry (MOI 10) for 4 h at 37 °C before fixation. Infected bacilli appear in red. These results were obtained at least two times. Scale bars, 30 µm. **f**, Flow cytometry data represented as dot plot show the percentage of bacilli infected with the BPs∆*33*HTH_HRM10 mCherry fluorophage relative to the study population. This assay was conducted at least twice.

**Extended Data Fig. 2:**
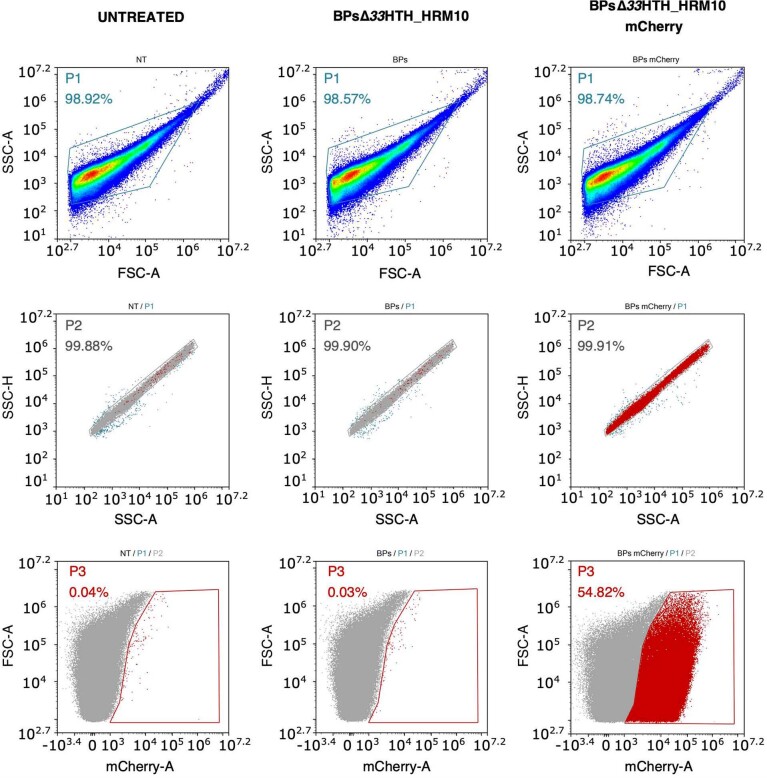
Gating strategy for flow cytometry analysis of fluorophage-infected bacteria. Bacteria untreated or infected with non-fluorescent phages were used as controls. The first gate was plotted on SSC-A / FSC-A to exclude cellular debris. SSC-H / SSC-A was analysed to eliminate multiple cells. The gate for fluorescence was drawn thanks to controls to exclude non-fluorescent bacteria. The analysis was performed on approximately 300,000 bacteria using NovoExpress software. Here is shown an example of analysis for GD22 strain performed at least twice.

**Extended Data Fig. 3:**
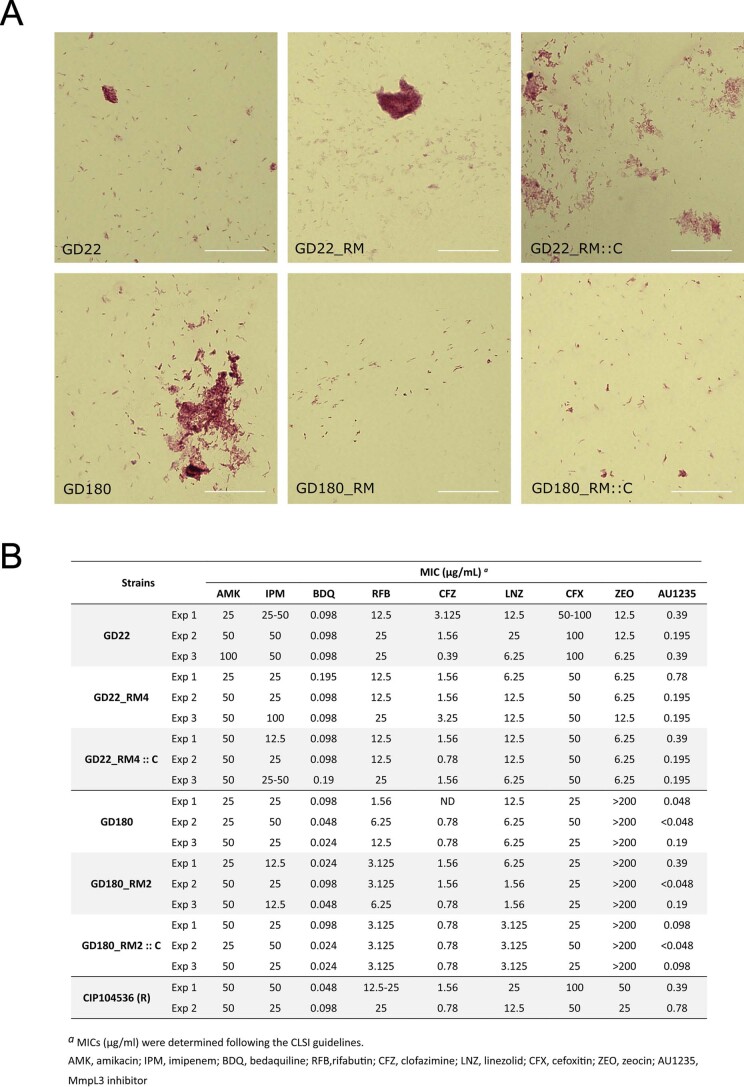
Characterization of TPP-defective mutants. **A**. Ziehl-Neelsen staining of *M. abscessus* TPP mutants. Cultures of GD22, GD22_RM, GD22_RM::C, GD180, GD180_RM and GD180_RM::C were fixed on glass slides and acid-fast staining was performed using the BD Carbolfuchsin kit, and observed microscopically at 60x magnification. This assay was performed three times. Scale bar: 30 µm. **B**. MIC (in µg/mL) values of antibiotics determined in Cation-adjusted Mueller-Hinton Broth (CaMHB) at 30 °C against *M. abscessus* clinical isolates (GD22 and GD180), resistant mutants (GD22_RM4 and GD180_RM2) and complemented strains (GD22_RM4::C and GD180_RM2::C) with CIP104536 (ATCC19977) as a control. Results from three independent experiments are shown.

Mutants GD22_RM4 and GD180_RM2, which are defective in *pks* and *mmpL10*, respectively (Fig. 4a), can both be complemented to fully restore BPsΔ*33*HTH_HRM10 and Muddy infection (Fig. 4b). Both mutants lack cell wall TPPs and TPPs are at least partially restored by complementation (Fig. 4c). Furthermore, a derivative of BPs expressing mCherry^35^, which behaves similarly to BPsΔ*33*HTH_HRM10 in plaque assays (Fig. 4b) and liquid infections (Fig. 4d), gives fluorescence from parent strains but not from GD22_RM4 and GD180_RM2 (Fig. 4e,f and Extended Data Fig. 2). Complementation fully restores liquid infection of GD180_RM2 and partially restores infection of GD22_RM4 (Fig. 4d), as well as fluorescence with the reporter phage (Fig. 4e,f). These data are consistent with an early defect in phage infection in these mutants, consistent with loss of adsorption to the cell surface. We note that disruption of TPP synthesis does not interfere with Ziehl-Neelsen staining of the bacteria or alter antibiotic sensitivities (Extended Data Fig. 3).

### *M. smegmatis* TPP mutants are resistant to BPs and Muddy

**Fig. 5:**
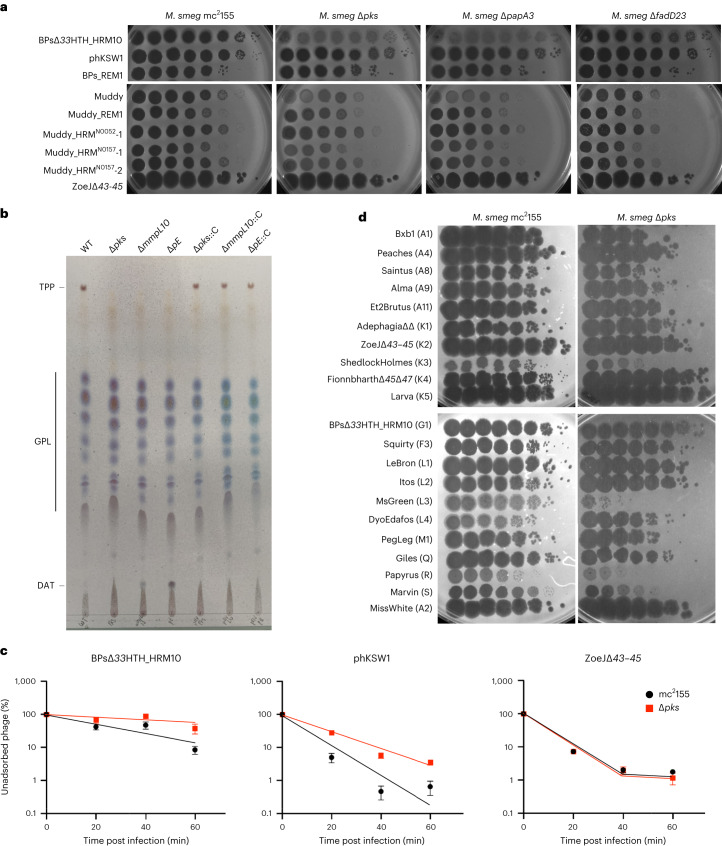
TPPs are also required for infection of *M. smegmatis* by phages BPs and Muddy. **a**, Tenfold serial dilutions of phages (as indicated on the left) were spotted onto solid media with *M. smegmatis* mc^2^155, ∆*pks*, ∆*papA3* or ∆*fadD23* as indicated. Plaque assays were performed at least twice with similar results. **b**, TLC analysis of total lipids extracted from wild-type *M. smegmatis*, three TPP-deficient mutants and the corresponding complemented strains. Eluent: CHCl_3_/CH_3_OH (90:10 v/v). TLC was revealed by spraying anthrone on the plate, followed by charring. **c**, Adsorption of phages BPs∆*33*HTH_HRM10, phKSW1 and ZoeJ∆*43–45* to *M. smegmatis* strains mc^2^155 and ∆*pks* as indicated by the percentage of unadsorbed phages remaining in the supernatant at different times after infection. Assays were performed in triplicate at least twice with similar results and a representative experiment is shown. Data are represented as mean ± s.d. Other replicates are shown in Extended Data Fig. 5. **d**, A panel of phages from various genetic clusters were tenfold serially diluted and spotted onto *M. smegmatis* mc^2^155 and ∆*pks*. Phage names are shown with their cluster/subcluster designation in parentheses. Plaque assays were performed twice with similar results.

**Extended Data Fig. 4:**
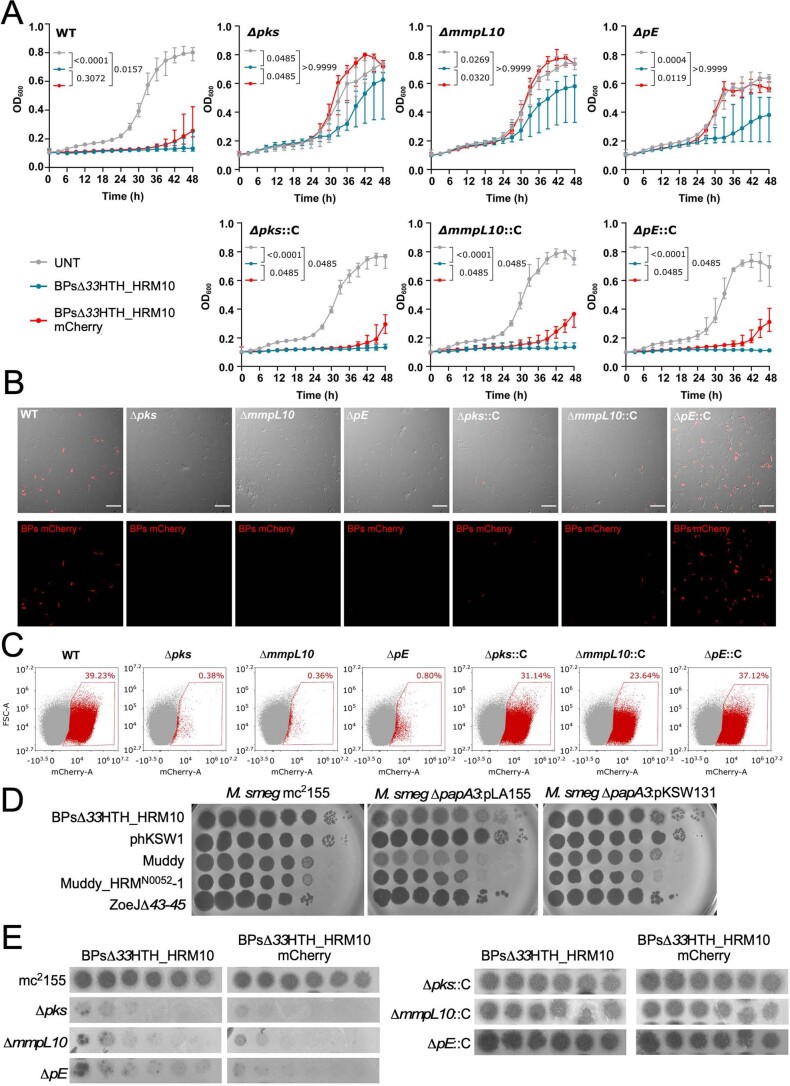
Infection of *M. smegmatis* TPP mutants by BPs and its derivatives. **A**. Growth curves of the different strains incubated with phage BPs∆*33*HTH_HRM10 or BPs∆*33*HTH_HRM10-mCherry at MOI 10 or without (UNT) for 2 days in 7H9/OADC supplemented with 1 mM CaCl_2_ at 37 °C without agitation. Measurements were taken every 3 hours. Data shown are represented as median of three independent experiments done in triplicate ± interquartile range. Two-sided Dunn’s multiple comparisons test was used to perform statistical analysis. Statistical analysis was done to compare the differences at 48 hours between each strain, p values are mentioned on each plot. **B**. Representative microscope fields of *M. smegmatis* strains infected with the fluorophage BPs∆*33*HTH_HRM10-mCherry (designated BPs mCherry) (MOI 10) for 2 hours at 37 °C. Similar results were obtained at least three times. Scale bars: 30 µm. **C**. Flow cytometry data plotted as a dot plot showing the percentage of bacilli infected with BPs∆*33*HTH_HRM10-mCherry relative to the study population. This assay was conducted twice with similar results obtained. **D**. Phage infection of *M. smegmatis* TPP mutants. Phages as shown on the left were tenfold serially diluted and spotted onto solid media with *M. smegmatis* mc^2^155, *M. smegmatis ∆papA3:*pLA155 or *M. smegmatis ∆papA3:*pKSW131. **E**. Plaquing of BPs∆*33*HTH_HRM10 and the mCherry derivative on *M. smegmatis* strains defective in TPP production (left panels) and the complemented strains (right panels). Phage lysates were tenfold serially diluted prior to spotting on bacterial lawns. Similar results were obtained at least three times and a representative experiment is shown.

*M. smegmatis* is genetically tractable and susceptible to a large number of diverse phages, and using TPP mutants in *pks (MSMEG_0408)*, *papA3* *(MSMEG_0409)*, *mmpL10 (MSMEG_0410)*, *fadD23* *(MSMEG_0411)* and *pE (MSMEG_0412)*^24,36^, we showed that these have similar, albeit somewhat milder, phenotypes to *M. abscessus* TPP mutants (Fig. 5a). As expected, Δ*pks*, Δ*mmpL10* and Δ*pE* mutants failed to produce TPPs, while complementation restores the presence of TPPs (Fig. 5b). The relatively efficient infection of the ∆*fadD23* mutant is consistent with incomplete TPP loss, possibly due to an unidentified fatty acyl-AMP ligase partially overcoming the defect^24^. Muddy similarly forms very turbid plaques on the Δ*pks* and Δ*papA3* mutants, but only mildly so on the Δ*fadD23* mutant (Fig. 5a). Interestingly, the TPP-independent BPs and Muddy mutants infect *M. smegmatis* TPP mutants normally (Fig. 5a). Complementation of the Δ*papA3*, Δ*pks*, Δ*mmpL10* and Δ*pE* mutants restores normal infection by both Muddy and BPsΔ*33*HTH_HRM10 (Extended Data Fig. 4).

**Extended Data Fig. 5:**
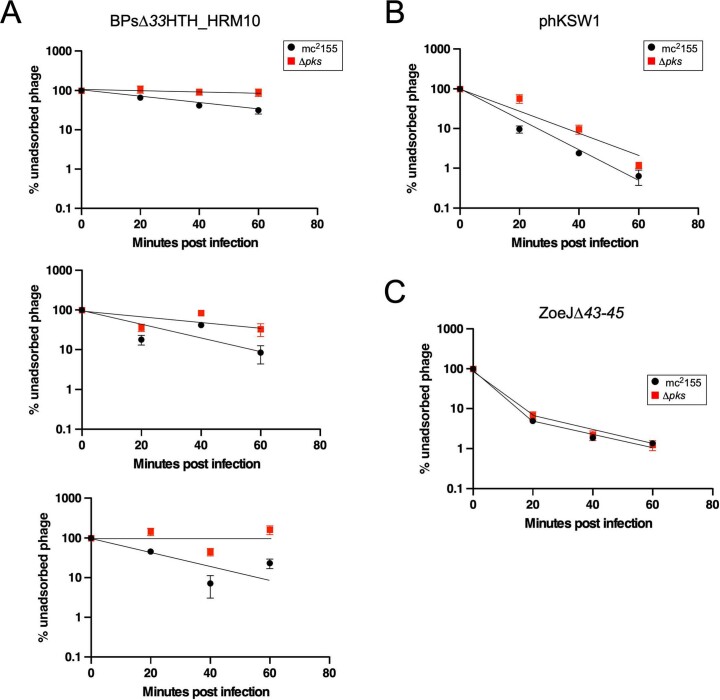
Additional replicates of phage adsorption assays on *M. smegmatis* mc^2^155 and ∆*pks*. Biological replicates of phage adsorption assays for phages BPs∆*33*HTH_HRM10 **(A)**, phKSW1 **(B)** and ZoeJ∆*43-45* **(C)** are shown. Data are represented as the mean of technical duplicates ± SD. For BPs∆*33*HTH_HRM10, each individual assay showed reduced adsorption on ∆*pks* as compared to mc^2^155. However, the measured adsorption rates varied between each replicate, such that observed differences were obfuscated when displaying the means; representative replicates for all phages are shown in Fig. 5.

BPsΔ*33*HTH_HRM10 and its mCherry derivatives are both defective in liquid infection of the Δ*pks*, Δ*mmpL10* and Δ*pE M. smegmatis* mutants, and efficient infection and lysis are restored by complementation (Extended Data Fig. 4a,e). The mCherry reporter phage shows fluorescence in wild-type *M. smegmatis* but loss of fluorescence in infection of all three mutants, with restoration of infection in the complemented strains (Extended Data Fig. 4b,c). In addition, the mCherry fluorophage behaves similarly to its parent in plaque assays on *M. smegmatis* mutant and complemented strains (Extended Data Fig. 4e). Both BPsΔ*33*HTH_HRM10 and the TPP-independent phKSW1 are defective in adsorption of a Δ*pks* mutant relative to wild-type *M. smegmatis* (Fig. 5c and Extended Data Fig. 5), similar to *M. abscessus* (Fig. 2b).

Testing a broader phage panel showed that most are not dependent on TPPs, except for ShedlockHolmes, MsGreen and Papyrus in Clusters/Subclusters K3, L3 and R, respectively, which show some TPP dependence (Fig. 5d). MsGreen and ShedlockHolmes have tail genes related to Muddy gene *24*, although we note that ZoeJ also does and yet is not TPP dependent. However, such variation is not unexpected, as the escape mutant observations show that only a single amino acid substitution is sufficient to confer TPP independence (Figs. 2a,c and 5a).

### TPP-independent phages reveal host resistance mechanisms

**Fig. 6:**
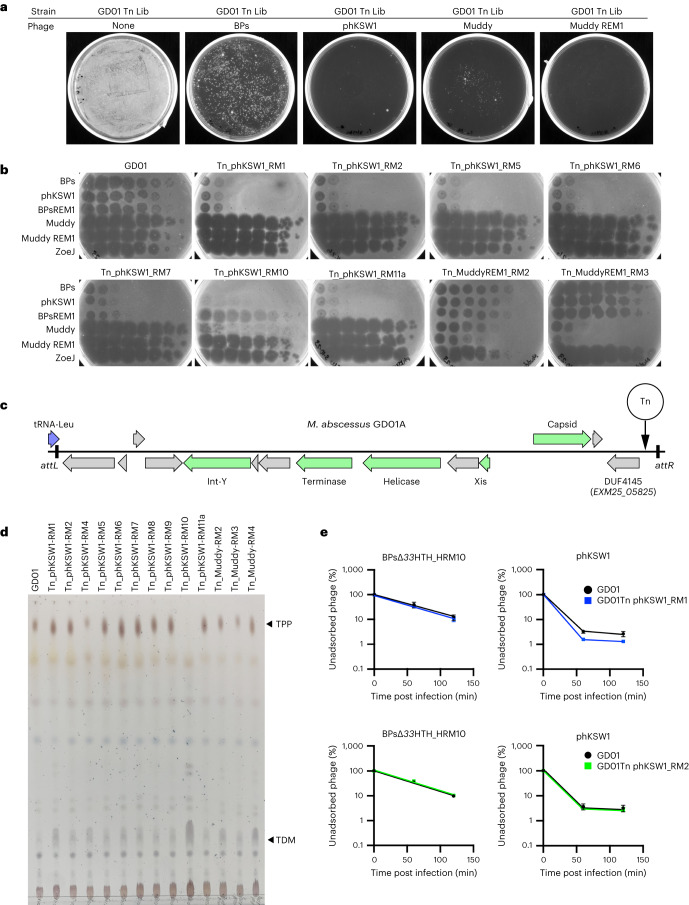
Evidence for additional phage resistance mechanisms. **a**, Recovery of *M. abscessus* GD01 transposon mutants resistant to phages BPs, phKSW1, Muddy and Muddy_REM1. A culture of an *M. abscessus* GD01 transposon library (GD01 Tn Lib) containing approximately 10^6^ c.f.u. was plated onto phage-seeded plates as indicated. **b**, Phage infection profiles of resistant mutants. Tenfold serial dilutions of phages BPsΔ*33*HTH_HRM10 (‘BPs’), phKSW1, Muddy, Muddy_REM1 and ZoeJΔ*43–45* (‘ZoeJ’) (as indicated on the left) were spotted onto lawns of *M. abscessus* GD01 or mutants isolated as resistant to TPP-independent phages phKSW1 and Muddy_REM1. Mutant designations indicate that they are Tn insertions (Tn), the phage used for mutant selection (that is, phKSW1 or Muddy_REM1) and the mutant number (for example, RM1, RM2 and so on). Where siblings are suspected (Extended Data Table 1), only one representative is shown; suspected siblings showed similar results. **c**, Organization of a putative candidate PICI in *M. abscessus* GD01. The satellite region is defined by the *attL* and *attR* attachment sites resulting from site-specific integration at a tRNA-Leu gene (GD01 coordinates 1158039 to 1170246). It contains several phage-related genes (green arrows) with the putative functions indicated. At the extreme right-hand end of the PICI, a gene (locus tag EXM25_05825) carries a DUF4145 domain implicated in phage defence in other systems. Four mutants contain a Tn insertion at the site, indicated by the vertical arrow. **d**, TLC analysis of total lipids extracted from *M. abscessus* GD01 and transposon mutants isolated as resistant to TPP-independent phages. **e**, Adsorption of BPsΔ*33*HTH_HRM10 and phKSW1 to GD01Tn_phKSW1_RM1 (blue, top panels) and to GD01Tn_phKSW1_RM2 (green, bottom panels). Adsorption to GD01 performed in parallel is shown in black. The proportions of phage particles remaining in solution are shown at different times after infection. Assays were performed in duplicate twice and data presented are mean ± s.d.

The TPP-independent phage mutants infect *M. abscessus* efficiently and we therefore repeated the selection for Tn insertion mutants to explore whether there are other surface molecules required for infection. Interestingly, such mutants arise from the same library at a 100-fold lower abundance than BPsΔ*33*HTH_HRM10 and Muddy resistant mutants (Fig. 6a and Extended Data Table 1). All but one of the phKSW1 resistant mutants analysed are similarly resistant to BPsΔ*33*HTH_HRM10, BPs_REM1 and phKSW1 but remain sensitive to Muddy and Muddy_REM1 (Fig. 6b); one (GD01Tn_phKSW1_RM10) is resistant to phKSW1 but is sensitive to BPs_REM1 and is resistant to Muddy but not Muddy_REM1 (Fig. 6b). One of the two Muddy_REM1 resistant mutants is only partially resistant to Muddy and Muddy_REM1, but both are fully sensitive to all of the BPs derivatives (Fig. 6b).

Characterization of these mutants shows that they are unlikely to be defective in surface recognition by the phages. Two Muddy_REM1 resistant strains have Tn insertions in transcription genes *greA* and *rpoC* (Extended Data Table 1), and one phKSW1 resistant mutant maps in *recB* (*MAB_0399c*; Extended Data Table 1); these are unlikely to be directly involved in phage binding. Four of the phKSW1 resistant mutants (RM2, RM5, RM7 and RM8), representing at least three independent insertions (Fig. 6 and Extended Data Table 1), have transposons at GD01 coordinate 1,169,901 in a region absent from ATCC19977 and many other *M. abscessus* strains and within a candidate ‘phage-inducible chromosomal island’ (PICI) (Fig. 6c and Extended Data Table 1). The insertions are upstream of GD01 gene *EXM25_05825* encoding a protein with a DUF4145 domain, which is implicated in a variety of viral defence systems and is often fused with restriction endonucleases and abortive infection systems^37–39^ (Fig. 6c). It is plausible that the BPs resistant phenotype results from overexpression of this gene.

Analysis of the cell wall lipids shows that all of the mutants retain TPPs, except for GD01Tn_phKSW1_RM10 (Fig. 6d), which has a Tn insertion in *papA3* in addition to a secondary insertion in *MAB_1686* (Extended Data Table 1). Two additional mutants (GD01Tn_phKSW1_RM1 and RM4) have an insertion in the nearby *MAB_1690* gene and have normal TPPs (Fig. 6d); *MAB_1686* and *MAB_1690* are within a large (22 kbp) operon encoding an Mce4 transport system (Extended Data Table 1). However, this Mce4 system is probably not acting as a receptor as the GD01Tn_phKSW1_RM1 mutant does not have an adsorption defect (Fig. 6e). The GD01Tn_phKSW1_RM2 mutant also does not have any adsorption defect (Fig. 6e).

## Discussion

Trehalose polyphleates are among the largest known lipids in mycobacteria and are structurally related to sulfolipids SL-1 and polyacylated trehalose PAT, which, in contrast to TPPs, are found exclusively in *M. tuberculosis*. The roles of TPPs in mycobacterial physiology and/or growth remain unclear, but they are implicated in clumping and cording in *M. abscessus*^25^. Many TPP-defective *M. abscessus* strains have rough morphotypes, typically associated with cording, consistent with the rough colony morphology primarily resulting from GPL loss^23^. Clearly, TPPs are critical for adsorption of several phages, including the therapeutically useful Muddy and BPs^4,5^. The finding that both phages require TPPs is a surprise, as they are genomically distinct, share few genes and were thus considered to be suitable for combination in phage cocktails. Nonetheless, the availability of TPP-independent phage mutants provides substitutes to which resistance to both phages occurs at a much lower frequency, and such mutants do not typically show co-resistance to the two phages. We propose that the TPP-independent phages replace their cognate parent phages in therapeutic cocktails.

A simple explanation for the role of TPPs is that they are specifically recognized and bound to by BPs, Muddy and the other TPP-dependent phages as the only requirement for DNA injection. However, it is then unclear as to how the TPP-independent phages overcome TPP loss, and it seems implausible that they gain the ability to bind to a completely different receptor. A more likely explanation is that TPPs act as co-receptors for Muddy and BPs and facilitate recognition of a different surface molecule; the TPP-independent phage mutants would then simply bypass the need for activation by TPPs. The observation that tail spike mutants such as phKSW1 adsorb substantially better than the parent phage, even to TPP-containing host cells, is consistent with this latter explanation. Furthermore, wild-type BPs does not efficiently infect *M. tuberculosis* H37Rv, but a mutant with the gp22 A604E substitution enables efficient infection^19^, even though *M. tuberculosis* lacks TPPs^40^. Similarly, wild-type Muddy efficiently infects *M. tuberculosis* H37Rv^20^ despite its lack of TPPs, and Muddy tail spike substitutions expand its host range to other *M. tuberculosis* strains^20^. These observations not only suggest that TPPs are not the receptors per se for these phages, but that there may be general mechanisms governing receptor access by the phages, together with phage strategies for expanding host cell recognition and infection. Furthermore, if TPPs are not the target of direct phage recognition, the true receptor is likely to be encoded by genes that are essential for mycobacterial viability. Thus, although transposon insertion mutagenesis has been used in other systems for identifying phage receptors^41–44^, this may be of more limited use in *Mycobacterium*, although as we have shown here, it is useful for mapping a plethora of resistance mechanisms.

Understanding the roles of TPPs in *M. abscessus* is important for therapeutic phage use, and we note that in at least some clinical isolates, the loss of TPPs through gene deletions or translocation leads to loss of infection by BPsΔ*33*HTH_HRM10 or Muddy. In the first therapeutic use of mycobacteriophages, BPsΔ*33*HTH_HRM10 and Muddy were used in combination with ZoeJ, and it is of interest that ZoeJ is not TPP dependent. We also note that resistance to BPs derivatives or Muddy has not been observed in clinical use, even in 11 cases where only a single phage was used^5^. It is plausible that resistance through TPP loss has a trade-off with fitness, although the roles of TPPs in *M. abscessus* pathogenicity and persistence are not known. Strikingly, the use of TPP-independent derivatives of BPs and Muddy not only avoids concerns about resistance via TPP loss, but also negates cross-resistance between the two phages (Fig. 6).

Finally, transposon mutagenesis and selection of mutants resistant to the TPP-independent phages reveal additional mechanisms of phage resistance. Particularly intriguing is the isolation of insertions in a candidate PICI, with the potential to activate expression of a PICI gene implicated in phage defence. These *Mycobacterium* PICIs and their roles in phage infection profiles deserve further investigation.

## Methods

### Bacterial strains and culture conditions

Bacterial strains (Extended Data Table 4) were grown in Middlebrook 7H9 media (BD Difco) supplemented with 10% oleic acid, albumin, dextrose and catalase (OADC enrichment) (7H9/OADC), or in Middlebrook 7H10/OADC solid media (BD Difco) at 37 °C. Antibiotics were added when required. Transformations of electrocompetent mycobacteria were performed using a Bio-Rad Gene pulser (25 µF, 2,500 V, 800 Ω). For some *M. smegmatis* strains, Tween80 (0.05%) was used in starter cultures but omitted in subcultures used for phage infections. Cultures used in phage infection were supplemented with 1 mM CaCl_2_. When required, *M. abscessus* strains were selected with 1 mg ml^−1^ hygromycin (Toku-E, 31282-04-9) or 200 µg ml^−1^ streptomycin, and *M. smegmatis* was selected with 50 µg ml^−1^ hygromycin. *M. abscessus* strains in the GDxx series are part of the strain collection at the University of Pittsburgh and were kindly provided by numerous colleagues.

### Engineering of MycoMarT7-Hyg2

Phage MycoMarT7-Hyg1 and MycoMarT7-Hyg2 were engineered from phage MycoMarT7 using CRISPY-BRED recombineering^31^. Briefly, double-stranded DNA recombineering substrates were designed that contained the desired mutation (a HygR cassette) with flanking sequences to permit the replacement of either KanR (MycoMarT7-Hyg1) or KanR and oriR6K (MycoMarT7-Hyg2). These substrates and genomic (g)DNA from MycoMarT7 were transformed into *M. smegmatis* mc^2^155 recombineering cells that contain the plasmid pJV138 (ref. ^31^). Transformations were combined with cells containing a CRISPR plasmid (a derivative of pIRL53) selecting against the parent MycoMarT7 and plated on solid media; this enriches for mutants containing the allelic replacement that form plaques on the plate. Resulting plaques were screened for the presence of the Hyg-marked transposon by PCR, and positive plaques were plaque purified, whole-genome sequenced and confirmed to have retained temperature sensitivity.

### Construction of the *M. abscessus* GD01 transposon insertion library and phage challenge

The transposon mutagenesis library was largely prepared as previously described^32^. Briefly, 50 ml of *M. abscessus* GD01 was grown to an OD_600_ of 0.2. The cells were pelleted and resuspended in 1 ml of phage buffer (10 mM Tris HCl (pH 7.5), 10 mM MgSO_4_, 68.5 mM NaCl, 1 mM CaCl_2_), pre-warmed to 37 °C and infected with 800 µl MycoMarT7-Hyg2 (5 × 10^10^ plaque-forming units (p.f.u.) ml^−1^). The cells and MycoMarT7-Hyg2 were incubated at 37 °C for 7.5 h. Cells were pelleted and resuspended in 8 ml PBS + 0.05% Tween80, and the resuspension was combined with 8 ml 40% glycerol for freezing at −80 °C. The transduction frequency was determined by measuring hygromycin-resistant colonies per ml and 120,000 transductants were plated onto large square plates containing solid 7H10/OADC media with 0.1% Tween80 and 1 mg ml^−1^ hygromycin. Plates were incubated at 37 °C for 9 d. To collect the library, cells were scraped off the solid media, resuspended in 7H9/OADC combined with 40% glycerol, aliquoted and frozen at −80 °C.

To identify GD01 insertion mutants that were resistant to phage infection, ~20 µl of GD01 Tn library was thawed and grown overnight to an OD_600_ of 0.175. Dilutions of this culture (approximately 10^4^, 10^5^ and 10^6^ cells) were spread onto 7H10/OADC solid media plates seeded with or without 10^8^ p.f.u. of phages BPs∆*33*HTH_HRM10, Muddy, phKSW1 or Muddy_REM1. Plates were incubated at 37 °C for 7 d. Colonies able to grow on phage-seeded plates were subjected to PCR to identify a transposon insertion site (see below) and struck out two times to remove any remaining phage. After streaking, single colonies were grown in liquid media and used for phage susceptibility testing by standard plaque assay.

### Identification of transposon insertion sites

Transposon insertion sites were identified by PCR using a primer that annealed to the transposon in the hygromycin resistance gene (Tn_Hyg_Fwd_2: 5′-CTTCACCTTCCTGCACGACT-3′) and a primer with a degenerate 3′ end, or if that did not yield an amplicon, nested PCR with primers Tn_Hyg_Fwd_2 and Primer 557 (5′-GGCCAGCGAGCTAACGAGCANNNNNNNGTT-3′) followed by PCR with primers Primer 414 (5′-GGCCAGCGAGCTAACGAGAC-3′) and Tn_Hyg_Fwd_1 (5′-TTCGAGGTGTTCGAGGAGAC-3′). Amplicons were gel extracted, Sanger sequenced from the transposon and the result aligned to the GD01 sequence to identify the transposon insertion site. For most strains (and at least one strain per interrupted gene), the transposon insertion site was confirmed by designing primers that flanked the site identified by the initial PCR and confirming that this region had increased in size by 1,259 bp compared with strain GD01. For five strains resistant to phKSW1 (GD01Tn_phKSW1_RM1, 2, 4, 6 and 10), the entire genomes were also sequenced as previously described^9^ (and see below) to confirm the location of inserted transposons. Reads were assembled into contigs and the location of the transposon sequence was identified. To confirm the total number of transposon insertions, reads at the transposon/chromosome boundaries were closely inspected to determine the number of branches, and the coverage of the transposon contig compared to the rest of the genome was determined. In the cases of RM1, 2 and 4, the transposon contig had approximately the same coverage as the rest of the genome and showed only one type of transposon/chromosome hybrid read at each end. In the cases of RM6 and 10, the transposon contig had approximately twice the coverage of the rest of the genome, and at each end, there were two types of transposon/chromosome hybrid read.

### Phages and screening of phage susceptibility

Phages used in this study were obtained from the University of Pittsburgh and *M. smegmatis* mc^2^155 was used to propagate them. Phage ZoeJ∆*43–45* is a derivative of the previously described ZoeJ∆*45* (ref. ^28^) and contains a deletion of genes *43* (integrase), *44* and *45* (repressor), corresponding to ZoeJ coordinates 33972–36489. It also contains the following single nucleotide polymorphisms different from ZoeJ: G3204T, A10165G, A10713G, C15262T. Phage susceptibility profiles were assessed using standard plaque assays. Top agar bacterial lawns were made by combining Middlebrook top agar (Middlebrook 7H9, 1 mM CaCl_2_, 0.35% BactoAgar) with 300–500 µl cell culture. After top agar had solidified, phages were tenfold serially diluted and spotted onto the top agar bacterial lawns and incubated for 24–48 h (*M. smegmatis*) or 5–7 d (*M. abscessus*) until bacterial lawns were confluent.

### Plasmid construction

To create plasmid pKSW131, *fadD23*, *pE*, *mmpL10* and *papA3* and the flanking intergenic sequence was amplified using Q5 HiFi 2× master mix (New England Biolabs) from gDNA isolated from *M. abscessus* GD01. The amplicon was purified and cloned into EcoRI-digested vector pLA155 using the NEBuilder HiFI DNA Assembly master mix (New England Biolabs) and transformed into *E. coli* strain DH5a; plasmids and primers are shown in Extended Data Table 5. The culture that yielded a successfully constructed plasmid was grown at 30 °C rather than 37 °C, although it is unknown whether this contributed to successful plasmid maintenance in the culture. To create plasmid pKSW134, the open reading frame of *fadD23* was amplified from GD01 gDNA and cloned into Pml I-digested anhydrotetracycline (ATc)-inducible vector pCCK39 (ref. ^45^) using the NEBuilder HiFI DNA Assembly master mix. The entire plasmids were sequenced using Plasmidsaurus (https://www.plasmidsaurus.com/).

pMV*pks*_*mWasabi* and pMV*mmpL10*_*mWasabi* were constructed on the basis of pMV*pks* and pMV*mmpL10* by in-fusion cloning. The *mWasabi* sequence under the control of the constitutive Pleft* promoter^46^ was amplified by PCR using a Q5 high-fidelity DNA polymerase (New England Biolabs). Plasmids were linearized with KpnI-HF (New England Biolabs). Agarose gels were used to purify linear fragments, then circularized using In-Fusion SNAP Assembly master mix (Takara) according to the manufacturer’s instructions. Stellar competent cells (Takara) were used for transformation. Plasmids generated were verified by sequencing (Eurofins Scientific).

### Isolation and whole-genome sequencing of phage-resistant mycobacteria mutants

Two of these phage-resistant mutants (GD17_RM1 and GD22 RM_4) were described previously^9^; the others were isolated in the same manner. Briefly, 10^8^ colony-forming units (c.f.u.) of *M. abscessus* were incubated with 10^9^ p.f.u. of phage. Infections were plated on solid media at 2 and 5 d post infection, and survivors were purified, tested for phage resistance and sequenced. The mutants were sequenced as described previously^9^. Briefly, 3 ml of culture was pelleted and resuspended in 600 µl nuclei lysis solution (Promega). Cells were added to lysing matrix B tubes (MP Biomedicals) and milled four times using a BeadBug6 microtube homogenizer (BenchMark). RNAse A (2 μl, Thermo Scientific) was added and the solution was incubated at 37 °C for 10 min. Phenol-chloroform-isoamyl alcohol was added to the lysed cells and the aqueous phase was removed after centrifugation. DNA was precipitated using isopropanol and 3 M sodium acetate, and washed two times with 75% ethanol before resuspension. Libraries were prepared with the NEBNext Ultra II FS Library Prep kit (New England Biolabs) and sequenced on an Illumina MiSeq. The resulting reads were aligned to the parent strain genome using Consed^47^. A custom programme (AceUtil) was used to identify differences between the mutant reads and the parent genome, and all mutations were confirmed by close inspection of the reads^48^.

### Isolation of BPs∆*33*HTH_HRM10 mutants

Clear plaques were observed within high-titre spots for phage BPs∆*33*HTH_HRM10 on strains GD01Tn_BPs_HRM10_RM6, GD01Tn_BPs_HRM10_RM11 and GD180_RM2. These plaques were picked and plated on the resistant strain two additional times to purify. A purified plaque was then used to produce a high-titre phage lysate on *M. smegmatis* mc^2^155 and subsequently subjected to gene *22* PCR sequencing or whole-genome sequencing.

### Total lipids extraction of mycobacteria and TLC analysis

Bacteria were grown in LB medium (Lennox, X964.3) at 37 °C without agitation and pelleted by centrifugation (3,000 *g*, 10 min, room temperature). Lipids were extracted from bacterial pellets treated successively with CHCl_3_/CH_3_OH (1:2) (Carlo Erba, 67-66-3; Honeywell, 67-56-1) and CHCl_3_/CH_3_OH (2:1), washed with water and dried. Lipids were resuspended in CHCl_3_ before spotting on TLC. For TLC analysis, silica gel G60 plates (10 × 20 cm, Macherey-Nagel) were used to spot samples and lipids were separated with CHCl_3_/CH_3_OH (90:10 v/v). Lipid profiles were shown by spraying the plates with a 0.2% anthrone (Sigma, 90-44-8) solution (w/v) in concentrated H_2_SO_4_ (Honeywell, 7664-93-9) and charring.

### Adsorption assays

*M. smegmatis* strains were grown to an OD_600_ of 0.5–0.8, then concentrated approximately tenfold to 1.75 × 10^9^ ml^−1^ in 7H9/10% albumin dextrose complex (ADC)/1 mM CaCl_2_. One millilitre of cells (*M. smegmatis* mc^2^155 or mc^2^155 ∆*pks*) was infected in triplicate in a 12-well plate at a multiplicity of infection (MOI) of 0.001. Cells were incubated at 37 °C with agitation. At each timepoint, 50 µl of liquid was removed and pelleted, and the supernatant that contained unbound phage was titred on *M. smegmatis*. For *M. abscessus* GD01 and *M. abscessus* GD01 *fadD23*::Tn, the same protocol was followed but the strains were grown to an OD_600_ of 0.15–0.25 and cells were concentrated approximately tenfold to 6.3 × 10^8^ ml^−1^ in 7H9/10% OADC/1 mM CaCl_2_.

### Growth curves of mycobacteria incubated with phages

Bacterial growth assays were performed in 96-well plates (Falcon), each well containing 100 µl of bacterial culture and 100 µl of phage lysates or medium as control. Exponential phage cultures of mycobacteria were used and set at 3 × 10^7^ c.f.u. ml^−1^ in Middlebrook 7H9/OADC supplemented with 1 mM CaCl_2_. Phages were incubated at an MOI of 10 and diluted in 7H9/OADC supplemented with 1 mM CaCl_2_. Measurements were taken every 3 h for *M. smegmatis* strains and every 6 h for *M. abscessus* strains using a spectrophotometer (Tecan, infinite 200 PRO) until stationary phase was reached (2 d for *M. smegmatis* strains and 6 d for *M. abscessus* strains). Plates were incubated at 37 °C without agitation.

### Microscopy and flow cytometry sample preparation

Mycobacteria were subcultured in 7H9/OADC with agitation to obtain exponential phase cultures. Bacteria were concentrated to obtain a sample containing 1.2 × 10^7^ c.f.u. (for microscopy) or 6 × 10^6^ c.f.u. (for flow cytometry) and then incubated with either medium or phage BPs∆*33*HTH_HRM10 (MOI 10) as controls or phage BPs∆*33*HTH_HRM10 mCherry (MOI 10). The infections were performed for 2 h and 4 h for *M. smegmatis* and *M. abscessus* strains, respectively, at 37 °C without agitation. After infection, samples were fixed with 4% paraformaldehyde (Electron Microscopy Sciences, EM-15714) for 20 min at room temperature. Samples were then diluted as necessary, depending on the experiment, with 7H9/OADC supplemented with 0.025% tyloxapol and sonicated to disrupt bacterial aggregates. For microscopy, samples were then mounted between coverslips and slides with Immu-Mount (Epredia). Samples were kept at 4 °C in the dark until analysis.

### Microscopy

Differential interference contrast and epifluorescence images were acquired on a ZEISS Axio Imager Z1 upright microscope. A ×63 Plan Apochromat 1.4 NA oil objective and a ×100 Plan Apochromat 1.4 NA oil objective were respectively used for *M. smegmatis* and *M. abscessus* strains. mCherry was excited with an Intenslight fibre lamp with Texas Red (Ex: 560/40, dic. 585, Em: 630/75) filter cube. Images were acquired with an scMOS ZYLA 4.2 MP camera.

### Image analysis

Representative fields without technical artefacts were chosen. Fiji software (version 1.53t) was used to adjust intensity, brightness and contrast (identically for compared image sets).

### Flow cytometry

Infected bacteria were analysed by flow cytometry using a NovoCyte ACEA flow cytometer (excitation laser wavelength: 561 nm, emission filter: 615/20 nm). Gates were drawn using SSC-A/FSC-A and multiple cells were excluded with SSC-H/SSC-A. Uninfected cells and bacteria infected by non-fluorescent phage were included as controls. Experiments were performed at least twice with similar results. Approximately 300,000 events were recorded per experiment. Analysis was done with NovoExpress version 1.6.1.

### Ziehl-Neelsen staining

Concentrated cultures were fixed on glass slides by heating at 150 °C for 15 min, followed by chemical fixation with methanol. BD Carbolfuchsin kit was used following the manufacturer’s instructions. Samples were observed using an Evos M7000 imaging system.

### Drug susceptibility testing

The Clinical and Laboratory Standards Institute guidelines^49^ were followed to determine the MICs. Briefly, all cultures were incubated in cation-adjusted Mueller–Hinton Broth (Merck, 90922) at 30 °C prior to the experiment. Each well of a 96-well plate was filled with 100 μl of bacterial suspension previously inoculated with 5 × 10^6^ c.f.u. ml^−1^, except for the first column, to which 198 μl of the bacterial suspension was added. Drug (2 μl) at its highest concentration was added to the first column containing 198 μl of bacterial suspension and was twofold serially diluted. Results were obtained after 4 d of incubation at 30 °C without agitation. Three independent experiments were carried out in duplicate.

### Statistical analysis

Statistical analysis was carried out with GraphPad Prism v.9.0.0 for Windows. Descriptive statistics are cited and represented as median and interquartile range for each of the variables calculated. A non-parametric Dunn’s test was used to compare the different conditions at 48 h for *M. smegmatis* or 144 h for *M. abscessus*. An a priori significance level was set at *α* = 0.05.

### Phylogenetic analysis of TPP pathway amino acid sequences in *M. abscessus*

A phylogenetic tree was constructed for a concatenated alignment of amino acid sequences of the five TPP synthesis pathway members for 143 clinical isolates of *M. abscessus* and *M. abscessus* ATCC19977. Homologues were identified using MMSeqs2 (v.13.45111) and phammseqs (v.1.0.4)^50^ and subsequently aligned using ClustalO (v.1.2.4) and Trimal (v.1.4.1)^51^. A concatenated alignment was generated with a custom Python script and the maximum-likelihood phylogeny was generated using RAxML (v.8.2.12)^52^.

### Reporting summary

Further information on research design is available in the Nature Portfolio Reporting Summary linked to this article.

### Supplementary information

Reporting Summary

## Figures and Tables

**Extended Data Table 1:**
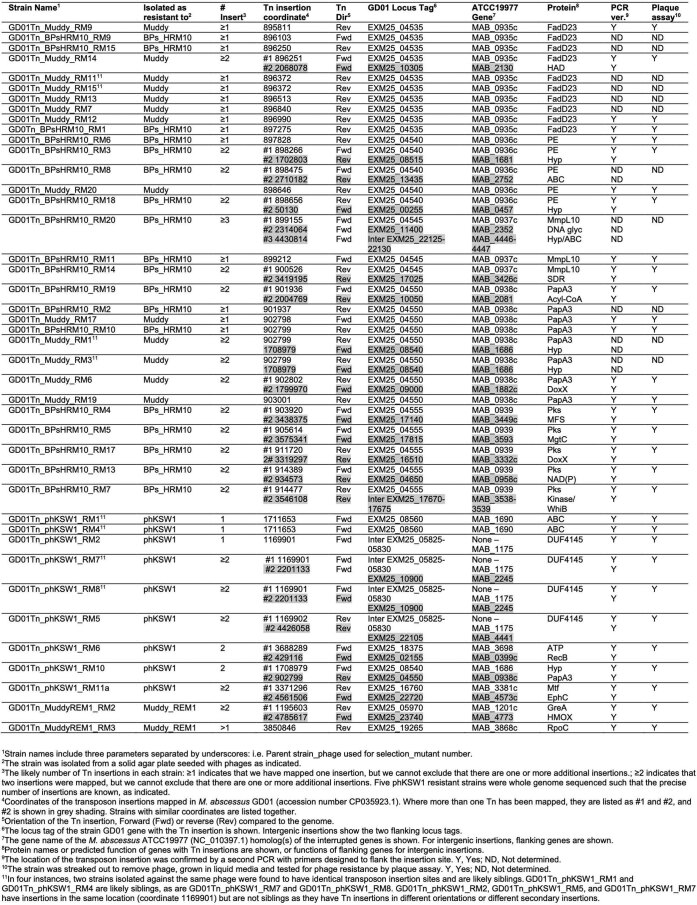
Characterization of phage resistant *M. abscessus* GD01 transposon mutants

**Extended Data Table 2:**
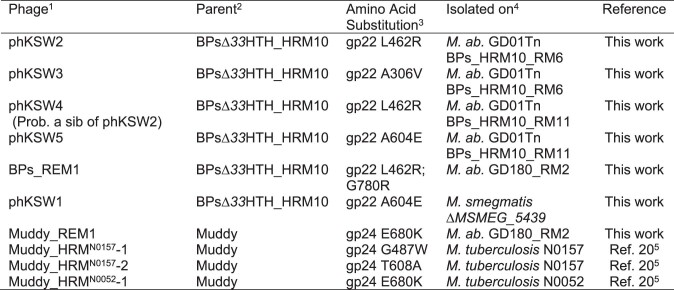
Phage BPs and Muddy mutants escaping TPP-loss mediated resistance

^1^Name of a phage able to form more clear plaques on TPP synthesis pathway mutants.

^2^Parent phage from which the mutant was isolated.

^3^Amino acid substitutions identified in mutant phages.

^4^The *M. abscessus* or *M. tuberculosis* strain on which the mutant phage was isolated

^5^Data from reference ^20^.

**Extended Data Table 3:**
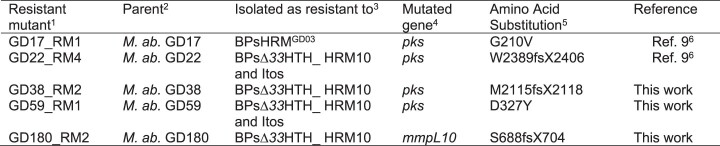
*M. abscessus* mutants spontaneously resistant to phage BPs derivatives

^1^Resistant mutants were named after the parent strain and the number of the resistant mutant (RM) isolated.

^2^Parent strain of *M. abscessus* from which the resistant mutant was isolated upon phage infection.

^3^RMs were isolated by infecting cell cultures at a high MOI and plating infections on solid media to isolate survivors.

^4^The entire genomes of the resistant mutants were sequenced and compared to the genomes of the parents to identify mutated genes conferring phage resistance.

^5^The amino acid substitution in the protein product resulting from the gene mutation.

^6^Data from reference ^9^.

**Extended Data Table 4:**
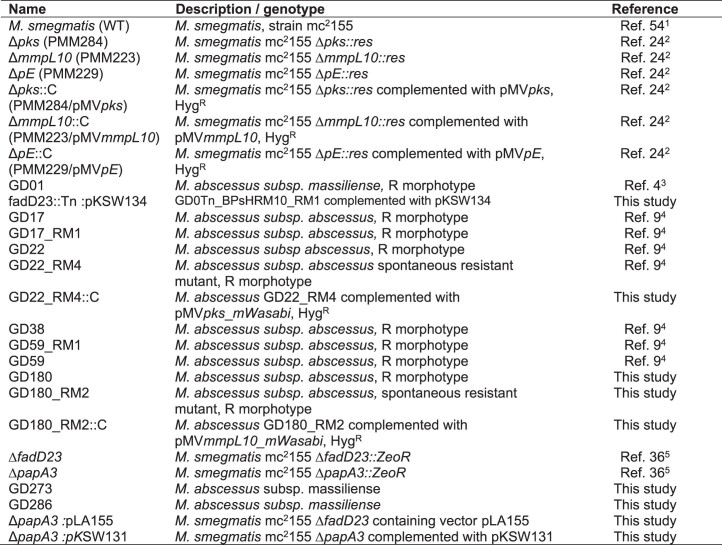
List of strains used in this study

^1^Data from reference ^54^.

^2^Data from reference ^24^.

^3^Data from reference ^4^.

^4^Data from reference ^9^.

^5^Data from reference ^36^.

**Extended Data Table 5:**
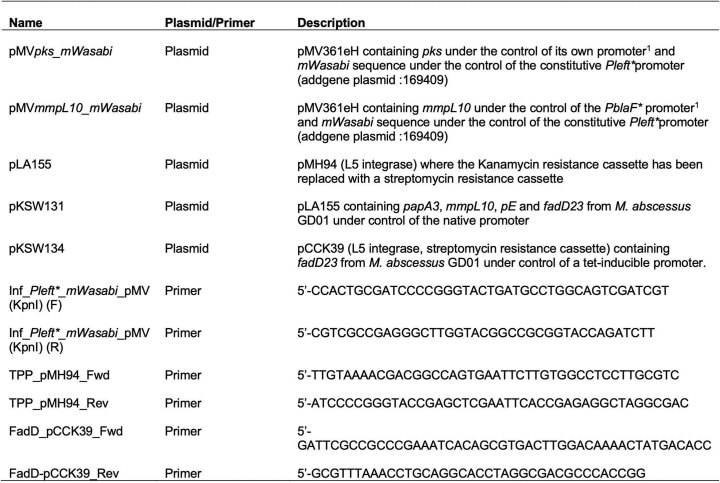
Plasmids and primers used in this study

## Data Availability

The genome sequences of mycobacteriophages referenced here are available at phagesdb.org. The genome sequences of the *M. abscessus* strains are available at https://osf.io/hjb7q/ and at NCBI BioProject PRJNA669041. All biological materials described in this study are available from G.F.H. at gfh@pitt.edu on reasonable request.
