# Supplementary material for: Therapeutically useful mycobacteriophages BPs and Muddy require trehalose polyphleates
Source: Nat Microbiol. 2023 Aug 24;8(9):1717–31. doi: 10.1038/s41564-023-01451-6 (PMC10465359; doi:10.1038/s41564-023-01451-6)
Supplement: Supplementary file 1 — Reporting Summary [file 41564_2023_1451_MOESM1_ESM.pdf]

nature portfolio

Corresponding author(s): Laurent Kremer
Graham Hatfull

Last updated by author(s): Jul 14, 2023

Reporting Summary

Nature Portfolio wishes to improve the reproducibility of the work that we publish. This form provides structure for consistency and transparency in reporting. For further information on Nature Portfolio policies, see our Editorial Policies and the Editorial Policy Checklist.

Statistics

For all statistical analyses, confirm that the following items are present in the figure legend, table legend, main text, or Methods section.

n/a	Confirmed	
☐	☒	The exact sample size (*n*) for each experimental group/condition, given as a discrete number and unit of measurement
☒	☐	A statement on whether measurements were taken from distinct samples or whether the same sample was measured repeatedly
☐	☒	The statistical test(s) used AND whether they are one- or two-sided<br>*Only common tests should be described solely by name; describe more complex techniques in the Methods section.*
☒	☐	A description of all covariates tested
☒	☐	A description of any assumptions or corrections, such as tests of normality and adjustment for multiple comparisons
☐	☒	A full description of the statistical parameters including central tendency (e.g. means) or other basic estimates (e.g. regression coefficient) AND variation (e.g. standard deviation) or associated estimates of uncertainty (e.g. confidence intervals)
☐	☒	For null hypothesis testing, the test statistic (e.g. F, t, r) with confidence intervals, effect sizes, degrees of freedom and P value noted<br>*Give P values as exact values whenever suitable.*
☒	☐	For Bayesian analysis, information on the choice of priors and Markov chain Monte Carlo settings
☒	☐	For hierarchical and complex designs, identification of the appropriate level for tests and full reporting of outcomes
☒	☐	Estimates of effect sizes (e.g. Cohen's *d*, Pearson's *r*), indicating how they were calculated

Our web collection on statistics for biologists contains articles on many of the points above.

Software and code

Policy information about availability of computer code

Data collection	Flow cytometry data was collected with NovoExpress.
Data analysis	Flow cytometry data was analyzed with NovoExpress. Fluorescent images were analyzed with Fiji. Graphs were generated and infection/adsorption data analyzed with GraphPad Prism 9.0. M. abscessus strain sequences were analyzed using MMSeqs2 (v13.45111) , phammseqs (v1.0.4), ClustalO (v1.2.4), Trimal (v1.4.1) and RAxML (v8.2.12). The concatenated amino acid alignment was generated with a custom Python script.

For manuscripts utilizing custom algorithms or software that are central to the research but not yet described in published literature, software must be made available to editors and reviewers. We strongly encourage code deposition in a community repository (e.g. GitHub). See the Nature Portfolio guidelines for submitting code & software for further information.

Data

Policy information about availability of data

All manuscripts must include a data availability statement. This statement should provide the following information, where applicable:

- Accession codes, unique identifiers, or web links for publicly available datasets
- A description of any restrictions on data availability
- For clinical datasets or third party data, please ensure that the statement adheres to our policy

Sequences of newly reported M. abscessus isolates are deposited in GenBank (accession numbers pending). All phage sequences are publicly available (phagesdb.org).

Human research participants

Policy information about studies involving human research participants and Sex and Gender in Research.

Reporting on sex and gender	N/A
Population characteristics	N/A
Recruitment	N/A
Ethics oversight	N/A

Note that full information on the approval of the study protocol must also be provided in the manuscript.

Field-specific reporting

Please select the one below that is the best fit for your research. If you are not sure, read the appropriate sections before making your selection.

☒ Life sciences ☐ Behavioural & social sciences ☐ Ecological, evolutionary & environmental sciences

For a reference copy of the document with all sections, see nature.com/documents/nr-reporting-summary-flat.pdf

Life sciences study design

All studies must disclose on these points even when the disclosure is negative.

Sample size	N/A
Data exclusions	No data exclusions.
Replication	All experiments that measured various steps in phage infection were performed in duplicate or triplicate, and performed at least twice. Similar results were found every time.
Randomization	N/A
Blinding	N/A

Reporting for specific materials, systems and methods

We require information from authors about some types of materials, experimental systems and methods used in many studies. Here, indicate whether each material, system or method listed is relevant to your study. If you are not sure if a list item applies to your research, read the appropriate section before selecting a response.

Materials & experimental systems

n/a	Involved in the study
☒	☐ Antibodies
☒	☐ Eukaryotic cell lines
☒	☐ Palaeontology and archaeology
☒	☐ Animals and other organisms
☒	☐ Clinical data
☒	☐ Dual use research of concern

Methods

n/a	Involved in the study
☒	☐ ChIP-seq
☐	☒ Flow cytometry
☒	☐ MRI-based neuroimaging

Flow Cytometry

Plots

Confirm that:

☒ The axis labels state the marker and fluorochrome used (e.g. CD4-FITC).

☒ The axis scales are clearly visible. Include numbers along axes only for bottom left plot of group (a 'group' is an analysis of identical markers).

☒ All plots are contour plots with outliers or pseudocolor plots.

☒ A numerical value for number of cells or percentage (with statistics) is provided.

Methodology

Sample preparation	M. abscessus or M. smegmatis cells were incubated with either medium or phage BPs_Δ33HTH_HRM10 as controls or phage BPs_Δ33HTH_HRM10 mCherry (MOI 10). After incubation, samples were fixed with PFA 4% for 20 min at room temperature. Samples were then diluted as necessary depending on the experiment with 7H9/OADC supplemented with 0.025% tyloxapol and sonicated to disrupt bacterial aggregates.
Instrument	NovoCyte ACEA flow cytometer
Software	NovoExpress
Cell population abundance	Approximately 300,000 events were recorded per experiment.
Gating strategy	Gates were drawn using SSC-A/FSC-A and multiples of cells were excluded with SSC-H / SSC-A.

☒ Tick this box to confirm that a figure exemplifying the gating strategy is provided in the Supplementary Information.
